# Lack of receptor for advanced glycation end products attenuates obesity-induced adipose tissue senescence in mice

**DOI:** 10.1080/21623945.2025.2611481

**Published:** 2026-01-09

**Authors:** Zuoqin Du, Jiaqi Wu, Tao Zhang, Xiaoyu Ma, Ziyu Li, Jin Xu, Jingcan You, Ni Chen, Jianbo Wu

**Affiliations:** a Department of Pharmacy, The Second People’s Hospital of Yibin, Yibin, China; b Basic Medicine Research Innovation Center for Cardiometabolic Diseases，Ministry of Education, Laboratory for Cardiovascular Pharmacology, Department of Pharmacology, School of Pharmacy, Luzhou Municipal Key Laboratory of Thrombosis and Vascular Biology Southwest Medical University, Luzhou, China

**Keywords:** Adipose tissue, receptor for advanced glycation end products (rage), senescence, oxidative stress, high-fat diet

## Abstract

The receptor for advanced glycation end products (RAGE) and its ligands are critical drivers of adipose tissue inflammation. While RAGE expression increases in ageing cells and pathological conditions, its specific role in high-fat diet (HFD)-induced adipose tissue senescence remains to be fully elucidated. In this study, we investigated the function of RAGE in the development of adipose tissue senescence associated with obesity. We observed that HFD-fed RAGE-deficient (RAGE^−/−^) mice exhibited significantly reduced body weight and adipocyte hypertrophy compared to wild-type (WT) controls. At the molecular level, RAGE^−/−^ mice displayed lower mRNA expression of cell cycle regulators and markers of the senescence-associated secretory phenotype. This anti-senescent phenotype was accompanied by decreased reactive oxygen species (ROS) production and elevated expression of anti-oxidant genes. Mechanistically, the lack of RAGE resulted in the upregulation of silent information regulator type 1 (SIRT1) in adipose tissues. Notably, the inhibition of SIRT1 reversed these anti-senescent effects and attenuated anti-oxidant gene expression in RAGE-deficient mice. Furthermore, while antioxidant treatment with N-acetylcysteine (NAC) reduced p53 in WT mice, it failed to fully suppress p16 and p21, whereas NAC treatment in RAGE^−/−^ mice significantly downregulated all senescence markers, suggesting a synergistic protective effect. In conclusion, our results demonstrated that RAGE deficiency improved anti-oxidant properties and prevents adipocyte senescence via the SIRT1 signalling pathway, highlighting a potential therapeutic target for obesity-associated tissue dysfunction.

## Introduction

Adipose tissue serves as an essential endocrine regulator in glucose homoeostasis and is involved in the progression of obesity and type 2 diabetes mellitus (T2DM). The cellular senescence in adipose tissue causatively induces defective adipogenesis, adipose tissue inflammation, and aberrant cytokine production, which links obesity to insulin resistance [1,2]. Advanced glycation end products (AGEs) interact with the receptor for advanced glycation end products (RAGE), expressed in adipocytes, to be involved in the progression of obesity, correlating with adipose tissue inflammation, adipocyte hypertrophy, and insulin sensitivity [3–5]. RAGE ligands such as HMGB1, is reported to associate with a set of pro-inflammatory processes and senescence [6].

Senescent cells gradually accumulate in adipose tissues during obesity, and insulin resistance is one of the consequences of adipose tissue senescence [7]. A previous report indicated that obesity causes adipose tissue dysfunction, correlated with adipocyte senescence, thus resulting in impaired adipogenesis and secretion of inflammatory cytokine [8]. These processes were mediated by the connection between NAD+ and SIRT1 in adipose tissues [9]. The senescent cell cycle arrest is mainly related to p16, p21, and p53. The age-dependent cellular senescence of adipose tissues has been linked to mitochondrial dysfunction and abnormal change in reactive oxygen species (ROS) [10–12]. Senescent cells and their secretory pro-inflammatory factors, the senescence-associated secretory phenotype (SASP), overlap with those secreted by dysfunctional adipose tissue. It has been revealed that RAGE ligands can induce macrophage activation and upregulation of inflammatory factors [13,14]. Inflammatory responses in adipose tissues are characterized by increased levels of oxidative stress, facilitating insulin resistance [15]. Our previous study has demonstrated that RAGE^−/−^ mice displayed improved inflammatory profiles and evidence for increased adipose tissue browning [5]. However, the potential role(s) of RAGE in obesity-associated oxidative stress and senescence remains unclear.

In obesity, the accumulation of AGE in adipose tissue is associated with increased tissue glycation, further contributing to diabetic complications [16]. Here, we aimed to study the relative roles of RAGE in analysing the effects of oxidative stress on adipose tissue senescence. We found that RAGE deficiency potently reduces the cellular senescence, SASP and increases anti-oxidant efficacy. These data demonstrate that the RAGE can impact cellular senescence and oxidative stress responses in adipose tissues.

## Materials and methods

### Animals

Six to eight-week-old wild-type (WT) mice were procured from Chongqing Medical University, Chongqing, China, while mice deficient in the receptor for advanced glycation end-products (RAGE^−/−^) were sourced from the Jackson Laboratory, Bar Harbor, ME, USA. Ethical oversight encompassed a thorough review and approval of all animal use protocols by the Animal Care Committee of Southwest Medical University, conducted in accordance with the guidelines set forth by the Institutional Animal Care and Use Committee.

### HFD-fed mouse model

Both wild-type (WT) and RAGE^−/−^ genotypes were subjected to a 14-week duration of high-fat diet (HFD) feeding (TP2330055A). The HFD was characterized by a composition of 60% fat, 25% carbohydrate, and 15% protein in caloric content, as provided by Research Diet, Trophic Animal Feed High-tech Co. Ltd, China, following previously outlined methodologies [5]. Control cohorts comprised age-matched male counterparts maintained on a standard chow diet (ND; TP2330055AC), with a nutritional profile consisting of 10% fat, 75% carbohydrate, and 15% protein in caloric content, from the same research diet source. This dietary manipulation served as a pivotal component of the investigative framework.

### Quantitative real-time PCR

Epdidymal adipose tissue (eAT) was collected and total RNA was extracted using TRIzol reagent (Invitrogen, Carlsbad, CA, USA). RNA samples were pre-treated with deoxyribonuclease I (Invitrogen Life Technologies, Carlsbad, CA, USA), and a SuperScript kit (Invitrogen Life Technologies, Carlsbad, CA, USA) was used to synthesize cDNA according to the manufacturer’s recommendations. qRT-PCR was analysed using miScript SYBR Green PCR Kits (Qiagen). Levels of SASP and oxidative stress markers mRNAs were determined using an ABI PRISM 7700 cycler (Applied Biosystems, Foster City, CA). Fold changes in gene expression were determined using the 2−ΔΔCT method. In some experiments, eAT was minced into approximately 1-mm pieces with 50 mg of eAT explants, and then was co-incubated with or without the SIRT1 inhibitor E×527 (Selleck) (10 μM) (10) or the ROS inhibitor N-acetylcysteine (NAC, Sigma, USA) (10 mM) for 24 h [17]. The values are presented as the mean ± SEM. All primers are listed in Supplemental Table S1.

### SIRT1 siRNA

The scramble and SIRT1 siRNA were purchased from RIBOBIO (Guangzhou, China). The SIRT1 siRNA target sequence: 5′- GGTTGTTAATGAAGCTATA −3′. eAT explants were transfected with 100 nM of siRNA using the RNAi transfect kit (RIBOBIO). Transfected explants were incubated in culture medium for 72 hrs and were harvested for detection of SIRT1 mRNA by qPCR. The oligonucleotide sequences of PCR primers were: mouse SIRT1 (TAGCCTTGTCAGATAAGGAAGGA, ACAGCTTCACAGTCAACTTTGT).

### Immunoblotting

Epididymal adipose tissue (eAT) lysates were methodically prepared, and equimolar quantities of protein were subjected to electrophoretic separation using sodium dodecyl sulphate-polyacrylamide gel electrophoresis (SDS-PAGE). Subsequent to electrophoresis, the proteins were transferred onto polyvinylidene difluoride membranes via electroblotting. Following this, membranes underwent blocking procedures, and immunoblotting was performed with antibodies specific to SIRT1 (Cell Signalling), p16 (Abcam), p21 (Servicebio), and p53 (Abcam). A secondary antibody, specifically horseradish peroxidase (HRP)-conjugated goat immunoglobulin G (IgG) raised against IgG (Santa Cruz Biotechnology), was employed. Blot visualization was accomplished through chemiluminescent detection using an enhanced chemiluminescence (ECL) substrate (Pierce).

### Senescence-associated-β-galactosidase (SA-β-gal) assay

The SA-*β*-gal activity was measured using Senescence Assay Kit (ST429, Beyotime) based on the manufacturer’s instructions. Briefly, eAT explants were incubated in ONPG at room temperature for 12 hours and then stained with the Staining Mixture at 37°C without CO_2_ overnight. Subsequently, eAT explants were observed and visualized under a light microscope (Zeiss HAL 100). The values were normalized to total protein levels assessed with a bicinchoninic acid (BCA) protein assay (Pierce).

### Tissue ROS levels

eAT was isolated, lysed, and the total amount of ROS was determined using the dihydroethidium (DHE) probe according to the manufacturer’s instructions (bjbalb Inc. Beijing, China). eAT was minced into approximately 1-mm pieces with 30 mg of explants, and added 1 mL of Buffer A for homogenization, which was centrifuged at 100 × *g*. Then add 200 μL of homogenate supernatant and 2 μL of DHE probe to 96-well plate for 30 min incubation. The fluorescence intensity was measured using 500 nm excitation wavelength and 610 nm emission wavelength. All values were normalized to total cellular protein, determined using a BCA assay, and expressed as intensity/mg protein.

### Immunohistochemistry

eAT was isolated, fixed, embedded in paraffin, and serially sectioned (6 µm). Cross-sections were prepared for immunohistochemistry by incubating overnight at 4°C with an antibody to p16 (Abcam), p21(Servicebio), p53 (Abcam), and γ-H2AX (Servicebio), respectively. The second antibody (HRP label) of the species corresponding to the first antibody was added at room temperature for 50 min. The images were captured using a microscope (Nikon). The percentage of positive cells/total adipocytes was quantified in five microscopic fields in each of the three cross sections of each tissue using ImagePro Plus software.

### Statistical analysis

Data are presented as the mean ± SEM of triplicate experiments. The significance of the differences among groups was analysed by one-way analysis of variance with a post hoc test to determine group differences in the study parameters. All analyses were performed with SPSS software (version 24.0 for Windows; Armonk, NY, USA), and a level of *p* < 0.05 was defined as indicative of statistical significance.

## Results

### Rage deficiency prevents the onset of senescence in adipose tissues

To define the role of RAGE in obesity *in vivo*, we fed mice a high-fat diet (HFD) for 14 weeks to induce obesity and hyperglycaemia. Under normal chow diet (NCD) conditions, we observed that RAGE^−/−^ mice exhibited significantly lower mRNA expression of cell cycle genes *p16*, *p21*, and *p53* in epididymal adipose tissue (eAT) compared to WT mice.

**Figure 1.**
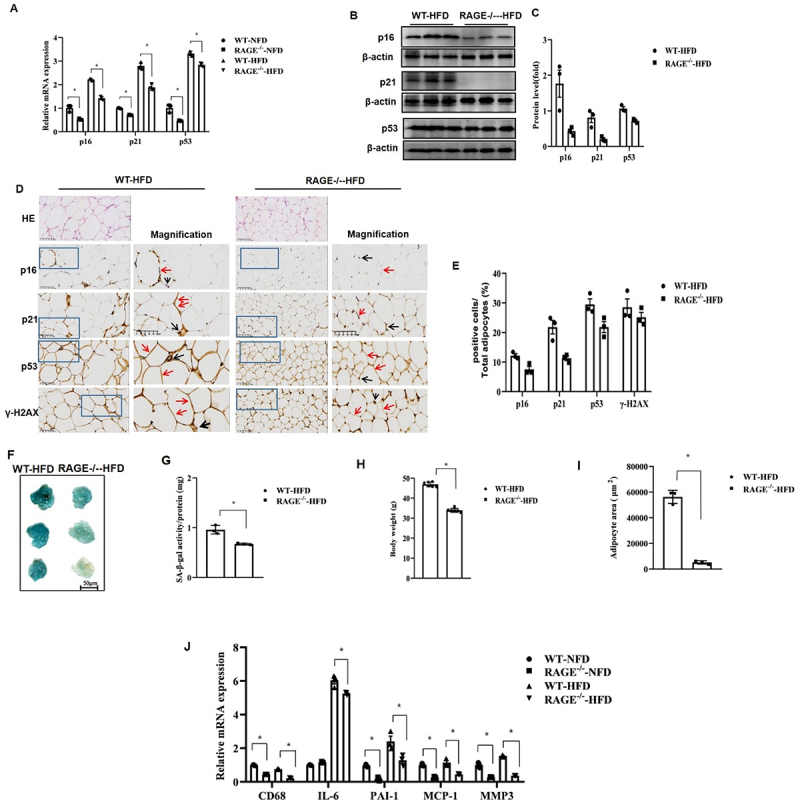
Rage deficiency prevents the onset of senescence in adipose tissues. (A) The expression of p16, p21, and p53 was evaluated by qPCR in eAT from normal chow diet (NCD)- or high-fat diet (HFD)-fed wt mice and rage-/- mice. Data are presented as mean ± SEM with individual data points overlaid (*n* = 6 per group). (B) p16, p21, and p53 protein levels measured by western blotting in eAT from WT-HFD and RAGE-/- HFD mice. (C) The graph corresponds to the adjacent blots and represents densitometric analyses. Data are shown as mean ± SEM with individual data points representing 3 independent experiments. *p < .05. (D) Eat sections were stained by hematoxylin and immunohistochemistry. Representative histological images were obtained from WT-HFD and RAGE-/- HFD mice. The boxes denote regions shown at a higher magnification in the adjacent column. Red arrows indicate positive adipocytes; black arrows indicate immune cells. Scale bar: 100 μm. (E) Quantification of p16, p21, p53, and $\gamma$-H2AX positive cells as a percentage of total adipocytes. Data are presented as mean ± SEM with individual data points superimposed. (F) Representative images of SA-β-galactosidase-positive eAT. (G) Senescence was evaluated in terms of SA-β-galactosidase activity and expressed as the ratio of tissue protein (mg). *n* = 6 per group. *p < .05. Data shown as mean ± SEM with individual points. Scale bar: 50 μm. (H) Body weight after 14 weeks of HFD feeding. (I) The area of adipocyte size. Data are presented as mean ± SEM (*n* = 6) with individual data points. *p < 0.05 vs. WT-HFD mice. (J) The mRNA levels of MCP-1, MMP3, IL-6, PAI-1, and CD68 of eAT quantified by qPCR. Data are presented as mean ± SEM with individual data points (*n* = 6). *p < 0.05 vs. WT-HFD mice.

HFD feeding markedly increased the mRNA levels of these markers in WT mice compared to the NCD group. A similar trend was observed in RAGE^−/−^ HFD mice compared to RAGE^−/−^ NCD mice. However, protein levels of p16, p21, and p53 were significantly lower in eAT from RAGE^−/−^ HFD mice compared to WT-HFD mice, as confirmed by Western blot analysis (Figure 1(B,C)).

Immunohistochemical staining revealed that while p53 and γH2AX were constantly present in adipocytes (red arrows) and some immune cells (black arrows), p16 and p21 were sparsely expressed in immune cells of WT-HFD mice compared to RAGE^−/−^ HFD mice (Figure 1(D,E)). To determine the senescent status of adipose tissue, we examined senescence-associated β-galactosidase (SA-β-gal) activity. Notably, adipose tissue from WT-HFD mice displayed significantly higher SA-β-gal activity compared to RAGE^−/−^ HFD mice (Figure 1(F,G)).

We also assessed phenotypic changes after 14 weeks of HFD feeding. WT-HFD mice displayed significantly higher body weight than RAGE^−/−^ HFD mice (Figure 1(H)). Furthermore, the epididymal adipocyte area was significantly decreased in RAGE^−/−^ HFD mice compared to WT-HFD mice (Figure 1(I)).

Since senescence is associated with a secretory phenotype (SASP), we confirmed by qPCR that RAGE deficiency under both NCD and HFD conditions resulted in decreased mRNA levels of specific SASP markers, including *PAI-1*, *MMP3*, *CD68*, *IL-6*, *MCP-1*, and *IGF-1* in eAT (Figure 1(J)). These findings demonstrate that RAGE plays an essential role in the onset of adipose tissue senescence during obesity.

### Rage deficiency prevents oxidative stress in adipose tissues

**Figure 2.**
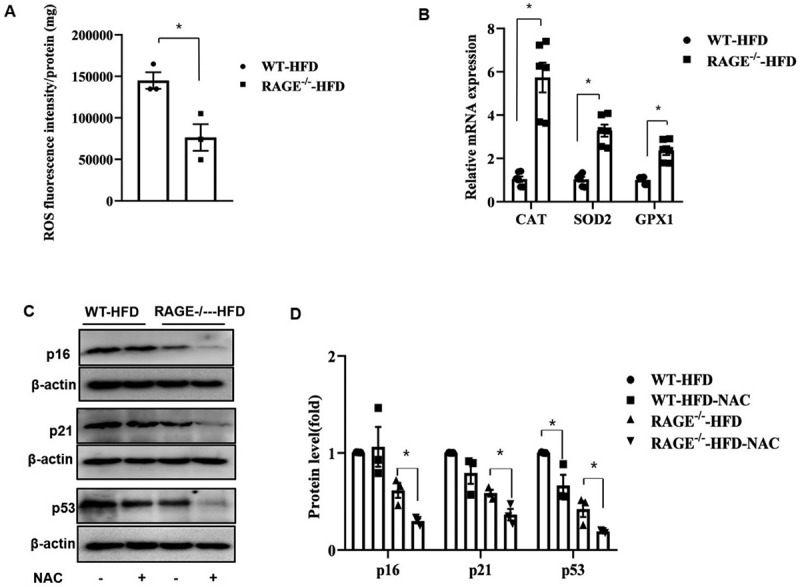
Rage deficiency prevents oxidative stress in adipose tissues. (A). The level of ROS production in eAT from HFD-fed wt mice and RAGE^−/−^ mice. (B). Cat, SOD2, and GPX1 mRNA expression levels in eAT of WT-HFD and RAGE^−/−^-HFD mice. (C). The expression of p16, p21, and p53 was evaluated by Westernbblotting in eAT in the presence or absence of NAC (10 mM) from WT-HFD and RAGE^−/−^-HFD mice. (D).The graph corresponds to the adjacent blots and represents densitometric analyses of 3 individual samples. *p < .05. All group data are shown as mean ± SEM.

Adipose oxidative stress is a major contributor to metabolic dysfunction and is critical in the progression of senescence [18,19]. To determine whether RAGE directly influences reactive oxygen species (ROS) production, we measured ROS levels in eAT. ROS production was significantly reduced in RAGE^−/−^ HFD mice compared to WT-HFD mice (Figure 2(A)).

We further examined the expression of antioxidant genes. As shown in Figure 2(B), RAGE^−/−^ HFD mice exhibited significantly increased mRNA levels of catalase (*CAT*), superoxide dismutase 2 (*SOD2*), and glutathione peroxidase 1 (*GPX1*) in eAT compared to WT-HFD mice.

Of note, treatment with the ROS inhibitor and antioxidant N-acetylcysteine (NAC) prevented RAGE-mediated senescence features in eAT. We evaluated the protein levels of p16, p21, and p53 by Western blot. As shown in Figure 2(C,D), while NAC inhibited p53 expression in eAT from WT-HFD mice, it did not significantly alter p16 and p21 levels. In contrast, in eAT from RAGE−/− HFD mice, NAC treatment significantly decreased the levels of p16, p21, and p53 compared to the control group. Collectively, these data indicate that the lack of RAGE directly affects obesity-related oxidative stress in adipose tissues.

### Rage deficiency prevents oxidative stress and senescence through SIRT1

**Figure 3.**
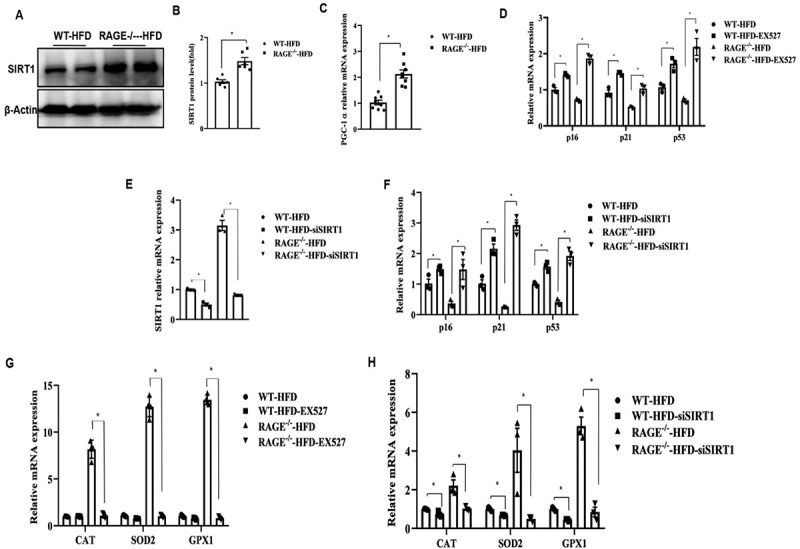
Rage deficiency prevents oxidative stress and senescence through SIRT1. (A). Representative immunoblots and quantification of eAT from WT-HFD and RAGE^−/−^-HFD mice as indicated. (B). The graph corresponds to the adjacent blots and represents densitometric analyses of 3 independent experiments. *p < .05. (C). The expression of PGC-1α was evaluated by qPCR from WT-HFD and RAGE^−/−^-HFD mice. (D). The treatment with SIRT1 inhibitor E×527 (10 μM) for 24 hours in eAT explants from WT-HFD and RAGE^−/−^-HFD mice. The total rna was extracted, and the expression of p16, p21, and p53 was evaluated by qPCR as indicated. (E, F). eAT explants were either transfected with the siRNA targeting SIRT1 (100 nM) or with scrambled siRNA duplex 100 nM) for 72 hrs and the mRNA expression levels of SIRT1 (E), p16, p21, and p53(F) were quantified by qPCR. (G). The treatment with E×527 for 24 hours in eAT explants from WT-HFD and RAGE^−/−^-HFD mice. The total rna was extracted, and the expression of cat, SOD2, and GPX1 was evaluated by qPCR as indicated. (H). Transfection of SIRT1 siRNA or scrambled siRNA for 72 hrs and the expression of cat, SOD2, and GPX1 was evaluated by qPCR as indicated. *n* = 6 per group. *p < .05. All group data are shown as mean ± SEM.

High glucose and AGEs are known to induce RAGE and downregulate SIRT1, a protein whose expression levels are linked to cellular senescence and oxidative stress [10,20–22]. We therefore examined whether RAGE plays a role in mediating SIRT1 expression in eAT. SIRT1 protein levels were found to be significantly higher in eAT from RAGE^−/−^ HFD mice than in WT-HFD mice (Figure 3(A,B)). Since SIRT1 regulates PGC-1α, we also evaluated PGC-1α expression. The results showed that PGC-1α mRNA levels were significantly higher in eAT from RAGE^−/−^ HFD mice compared to WT-HFD mice (Figure 3(C)).

To further examine the role of SIRT1 signalling in regulating senescence genes, we utilized the SIRT1 inhibitor EX-527 (Figure 3(D)) and SIRT1 siRNA (Figure 3(E,F)). Inhibition of SIRT1 significantly increased the expression of senescence genes in eAT from both RAGE^−/−^ HFD and WT-HFD mice, indicating a role for RAGE-SIRT1 signalling in the induction of senescence gene expression.

Given that RAGE is strongly associated with oxidative stress in eAT, we investigated whether SIRT1 is involved in the RAGE-mediated regulation of antioxidant genes. Consistent with our previous findings, treatment with EX-527 or SIRT1 siRNA attenuated the expression of CAT, SOD2, and GPX1 in eAT from RAGE^−/−^ HFD mice compared to WT-HFD mice (Figure 3(G,H)). Collectively, these results suggest that RAGE deficiency protects adipose tissues from oxidative stress and senescence via the activation of SIRT1.

## Discussion

The present study described a previously unrecognized role of RAGE in obesity-mediated adipose tissue oxidative stress and senescence. Our data suggest that RAGE^−/−^ mice exhibited reduced expression levels of mRNA related to cell cycle, cellular senescence markers, and senescence-associated secretory phenotype (SASP) following HFD-induced obesity. Further, RAGE deficiency decreased the production of ROS and increased the expression of anti-oxidant genes in adipose tissue. However, an intriguing aspect of our study is the observation that prevention of oxidative stress and senescence by RAGE deficiency, while involving SIRT1, is mediated by mediating senescent genes. AGE-RAGE interaction can change cell signalling, promote oxidative stress generation, and release pro-inflammatory molecules [21–23]. Beyond the molecular markers of senescence, we observed significant phenotypic changes. WT-HFD mice displayed significantly increased body weight and epididymal adipocyte area compared to RAGE^−/−^ mice. Obesity typically causes adipose tissue dysfunction correlated with adipocyte senescence, resulting in impaired adipogenesis and inflammatory cytokine secretion. The reduction in adipocyte hypertrophy in RAGE^−/−^ mice suggests that preventing senescence may preserve functional adipogenesis and lipid handling, thereby mitigating the gross morphological changes associated with HFD.

A previous study demonstrated that RAGE is required to impair p53 expression and p53 function in regulating p21 expression, involving a direct binding of RAGE to p53 in senescent preadipocytes [24]. We found a substantial decrease in ROS production in adipose tissues from RAGE-deficient mice. Previous studies have reported that oxidative stress induces senescence-like features and impairs metabolic functions in adipocytes. To interrogate this mechanism, we utilized the ROS inhibitor N-acetylcysteine (NAC). Interestingly, while NAC inhibited p53 expression in WT-HFD mice, it did not significantly reduce p16 and p21 levels. In contrast, NAC treatment in RAGE^−/−^ mice significantly decreased p16, p21, and p53. This discrepancy suggests a ‘threshold effect’: the oxidative and inflammatory burden in WT-HFD mice may be too high for NAC alone to reverse the established senescent phenotype, particularly the p16/p21 pathways. However, in RAGE^−/−^ mice, where basal oxidative stress is lower and antioxidant enzymes (CAT, SOD2) are naturally higher, the tissue is more responsive to antioxidant intervention, leading to a complete suppression of senescence markers

In obese, SIRT1 expression in adipose tissue was significantly suppressed, and downregulation of SIRT1 May contribute to obesity-associated metabolic abnormalities [25,26]. SIRT1 upregulates the activity of the antiaging protein in adipose tissues [27]. The knockdown of SIRT1 increased the expression of p16 and p21 in vitro [28,29]. SIRT1 binds and deacetylates p53 of modulating cellular senescence [30]. Our findings indicate that the prevention of oxidative stress and senescence by RAGE deficiency is mediated by SIRT1. In obesity, SIRT1 expression in adipose tissue is significantly suppressed. We observed that RAGE deficiency restored SIRT1 levels and that inhibiting SIRT1 (via EX-527 or siRNA) abolished the anti-senescent and anti-oxidant benefits observed in the RAGE^−/−^ mice. This supports the hypothesis that RAGE promotes senescence by interfering with the SIRT1/p53/p21/p16 signal axis.

We found a substantial decrease in ROS production in adipose tissues from RAGE deficiency mice, and further studies revealed RAGE-mediated antioxidant genes, leading to the anti-senescence for adipocytes. Previous studies reported that oxidative stress induced senescence-like features and impaired metabolic functions in adipocytes [17,31,32]. While our results identify RAGE as a key modulator of senescence, there are limitations to this study. We utilized a whole-body RAGE knockout model. Our immunohistochemical data showed p16, p21, p53, and γH2AX staining in both adipocytes and immune cells. Consequently, we cannot strictly rule out that the observed systemic benefits are partially mediated by immune cell modulation rather than exclusively by cell-autonomous adipocyte signalling. Future studies utilizing adipocyte-specific RAGE knockout models or primary adipocyte cultures are necessary to definitively isolate the cell-autonomous contributions of RAGE to adipocyte senescence.

**Figure 4.**
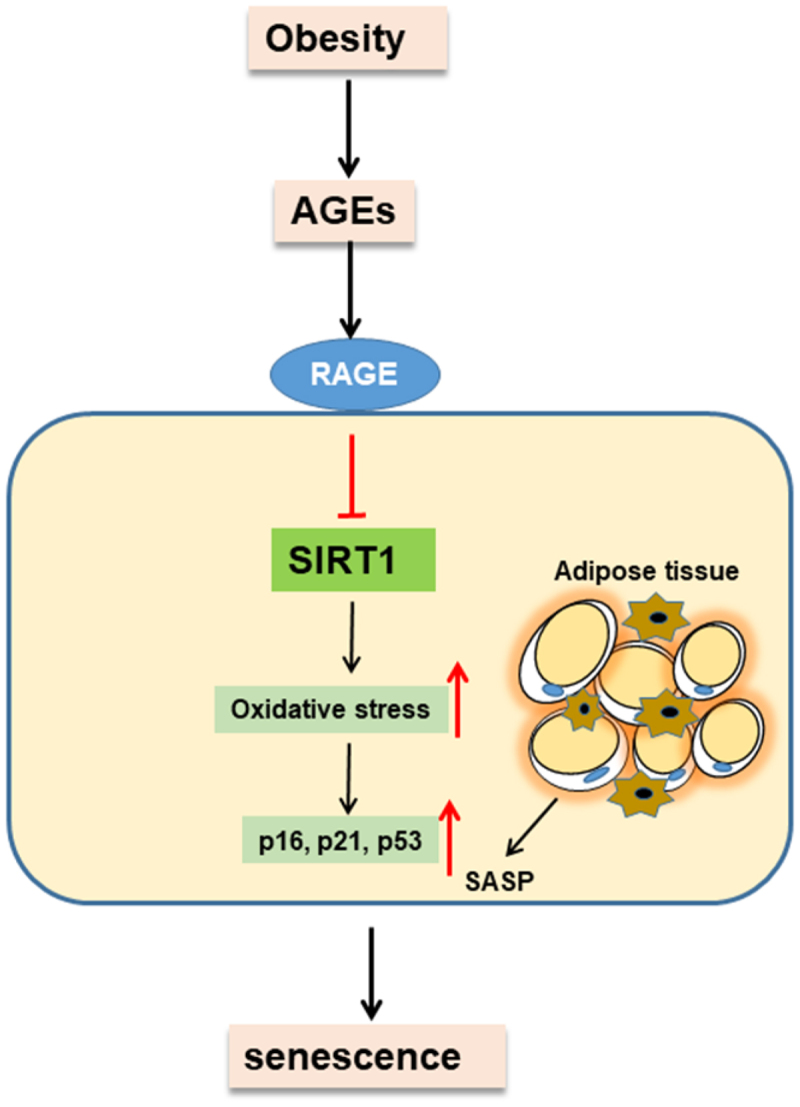
Schematic illustration of the possible signalling cascade through which rage regulates obesity-mediated adipose tissues senescence. rage deficiency promotes Sirtuin 1 (SIRT1) expression by downregulating oxidative stress, p16/p21/p53, and SASP and protected cell senescence.

In summary, we confirmed that RAGE regulates adipose tissue oxidative stress mediated by SIRT1. We propose that RAGE promotes senescence by interfering with the SIRT1 signalling axis and inducing oxidative stress, as illustrated in our proposed model (Figure 4). These findings suggest that targeting the RAGE-SIRT1 axis could be a viable strategy to ameliorate obesity-related adipose tissue ageing and dysfunction.

## Supplementary Material

Table of pimers.docx

## Data Availability

The data generated or analysed during this study are included in this article, or if absent are available from the corresponding author upon reasonable request.

## Abbreviations

SIRT1: Silent information regulator type 1
RAGE: Receptor for advanced glycation end products
ROS: Reactive oxygen species
SASP: Senescence-associated secretory phenotype
EAT: Epididymal adipose tissue
HFD: High-fat diet
NCD: Normal chow diet
SA-*β*-gal: Senescence-Associated-*β*-Galactosidase
