# Supplementary material for: Lack of receptor for advanced glycation end products attenuates obesity-induced adipose tissue senescence in mice
Source: Adipocyte. 2026 Jan 9;15(1):2611481. doi: 10.1080/21623945.2025.2611481 (PMC12795267; doi:10.1080/21623945.2025.2611481)
Supplement: Table of pimers.docx [file KADI_A_2611481_SM4411.docx]

**Primers used in qPCR**

| **Gene** |  | **Forward** | **Reverse** |
| --- | --- | --- | --- |
| 18S |  | CCTGGATACCGCAGCTAGGA | GCGGCGCAATACGAATGCCCC |
| P21 |  | ACTTCCTCTGCCCTGCTGC | GGTCTGCCTCCGTTTTCG |
| P16 |  | ATGGAGTCCGCTGCAGACAG | ATCGGGGTACGACCGAAAG |
| P53 |  | CCCCAGGATGTTGAGGAGTT | TTGAGAAGGGACAAAAGATGACA |
| MCP-1 |  | ATGCAGGTCCCTGTCATG | GTTCACTGTCACACTGGTCA |
| CD68 |  | CTAGTCCAAGGTCCAAGGGG | TCCCTGGACCTTGGTTTTGT |
| MMP3 |  | CCTCTATGGACCTCCCACAGAATC | GTGCCAATGCCTGGAAAGTTC |
| PAI-1 |  | CTATGGTGAAACAGGTGGACTT | GAACTTAGGCAGGATGAGGAG |
| IL-6 |  | CACATGTTCTCTGGGAAATCG | TTGTATCTCTGGAAGTTTCAGATTGTT |
| CAT |  | TTACCCCAACAGCTTCAGCG | GTCCGCACCTGAGTGACATT |
| SOD2 |  | GAGAACCCAAAGGAGAGTTGC | CTTATTGAAGCCAAGCCAGCC |
| GPX1 |  | TTCGGACACCAGGAGAATGG | TAAAGAGCGGGTGAGCCTTC |
| PGC1α |  | TATGGAGTGACATAGAGTGTGCT | CCACTTCAATCCACCCAGAAAG |
